# Quantitative proteomics approach reveals novel biomarkers and pathological mechanism of keloid

**DOI:** 10.1002/prca.202100127

**Published:** 2022-04-26

**Authors:** Jian Liu, Chunhua Yang, Huayu Zhang, Wei Hu, Jonas Bergquist, Helen Wang, Tingzhi Deng, Xueling Yang, Chao Zhang, Yanping Zhu, Xiaodong Chi, Jia Mi, Yibing Wang

**Affiliations:** ^1^ Department of Plastic Surgery, Shandong Provincial Qianfoshan Hospital Cheeloo College of Medicine, Shandong University Jinan Shandong China; ^2^ Department of Plastic Surgery The First Affiliated Hospital of Shandong First Medical University & Shandong Provincial Qianfoshan Hospital Jinan Shandong China; ^3^ Jinan Clinical Research Center for Tissue Engineering Skin Regeneration and wound Repair Jinan Shandong China; ^4^ Shandong Technology Innovation Center of Molecular Targeting and Intelligent Diagnosis and Treatment, School of Pharmacy Binzhou Medical University Yantai Shandong China; ^5^ Department of Chemistry – BMC, Analytical Chemistry and Neurochemistry Uppsala University Uppsala Sweden; ^6^ Department of Medical Biochemistry and Microbiology, BMC Uppsala University Uppsala Sweden

**Keywords:** bioinformatics, keloid, PDGFRL, proteomics, XBP1

## Abstract

**Background:**

Keloid is a pathological skin scar formation with complex and unclear molecular pathology mechanism. Novel biomarkers and associated mechanisms are needed to improve current therapies.

**Objectives:**

To identify novel biomarkers and underlying pathological mechanisms of keloids.

**Methods:**

Six pairs of keloid scar tissues and corresponding normal skin tissues were quantitatively analyzed by a high‐resolution label‐free mass spectrometry‐based proteomics approach. Differential protein expression data was further analyzed by a comprehensive bioinformatics approach to identify novel biomarkers and mechanistic pathways for keloid formation. Candidate biomarkers were validated experimentally.

**Results:**

In total, 1359 proteins were identified by proteomic analysis. Of these, 206 proteins exhibited a significant difference in expression between keloid scar and normal skin tissues. RCN3 and CALU were significantly upregulated in keloids. RCN1 and PDGFRL were uniquely expressed in keloids. Pathway analysis suggested that the XBP1‐mediated unfolded protein response (UPR) pathway was involved in keloid formation. Moreover, a *PDGFRL* centric gene coexpression network was constructed to illustrate its function in skin.

**Conclusions and Clinical Relevance:**

Our study proposed four novel biomarkers and highlighted the role of XBP1‐mediated UPR pathway in the pathology of keloids. It provided novel biological insights that contribute to develop novel therapeutic strategies for keloids.

## 1 INTRODUCTION

Keloid scars are pathological fibro‐proliferative disorders of the skin that exhibit abnormal phenotypes including fibroblast proliferation and collagen deposition [1]. There are several treatments for keloids including conventional surgical therapies and adjuvant therapies; however, a high recurrence rate of keloids is routinely observed after treatment [2]. Therefore, an improved understanding of the pathogenesis leading to keloid formation is needed to be able to develop novel targeted therapies. Cytokines and various growth factors are reported to be involved in keloid pathogenesis [1, 3]. For example, platelet derived growth factor (PDGF) regulates the production of collagen and fibronectin (FN), thus contributing to wound healing [4, 5, 6]. The abnormal activation of PDGF receptors and insulin‐like growth factor‐1 (IGF‐1) receptors induced by transforming growth factor‐β (TGF‐β) is known to be associated with the regulation of numerous processes in the skin regeneration pathway [7, 8, 9]. However, the overall pathogenic process underlying the development of keloids is still unclear and novel markers for treatment are urgently required.

Quantitative proteomics approaches have been proved to be an efficient approach for the investigation of pathological mechanisms and novel biomarkers. To date, a few proteomics studies have been conducted on keloid scars and some biomarkers of keloid scars have been suggested [10, 11, 12]. However, a more comprehensive and in‐depth analysis of keloid scars is still needed to reveal further details relating to the pathological mechanisms associated with keloids. The development of high‐resolution mass spectrometry and its combination with a state‐of‐the‐art bioinformatics approach provide us with an exciting option for investigative research. We have previously proposed several novel biomarkers and pathways for a variety of tumors through our proteomics platform [13, 14, 15, 16]. In this study, we present a label‐free quantitative proteomics analysis to explore differential protein expression profiles in normal skin and keloid scar tissues based on nano‐liquid chromatography and tandem mass spectrometry (nano‐LC–MS/MS). The expression profiling was further bioinformatically analyzed to identify the key regulators and pathways involved in keloid formation. Our findings provide a more comprehensive expression landscape of keloid proteins and yield novel pathological insights into the formation of keloid scars.

## 2 MATERIALS AND METHODS

### 2.1 Patients and tissue samples

Keloid scar tissues and normal skin tissues adjacent to the keloid scars were prospectively collected from six patients that were enrolled in Shandong Qianfoshan Hospital, Jinan, China. The patients did not receive any previous treatment for keloid scarring prior to surgical excision, and the details of the patients and tissues are described in Supporting Information Table S1. The samples were rinsed with PBS and snap frozen in liquid nitrogen immediately after surgical excision and stored at −80°C until analysis. This study was conducted in accordance with the Declaration of Helsinki and was approved by the ethical review board of Binzhou Medical University.

### 2.2 Protein extraction

The specimen was homogenized in lysis buffer (20 mM HEPES, 9 M Urea, EDTA‐free Protease Inhibitor Cocktail) and incubated for 30 min on ice. Then, the sample was sonicated with an ultrasonication probe (10 × 1 s) and centrifuged at 13,400 rpm for 10 min at 4°C. Supernatant was collected and the protein concentration of the protein extract was determined by Bradford assay (TaKaRa, Dalian, China) according to the manufacturer's instructions.

**Clinical Relevance**

Keloid scars can cause pain and in severe cases, ulcers and reduced joint mobility, thus leading to both physical and psychological distress for the affected individual. Keloid scars have a high recurrence rate under current therapies. The exact etiology of keloids remains unknown to a large extent. As an efficacious tool for mechanism research, proteomics approach is still limit in the application of research about keloid formation. In the present study, we present a comprehensive label‐free quantitative proteomics analysis of keloid scar and corresponding normal skin tissues from six patients, followed by further bioinformatic analysis. Our findings provide a more comprehensive understanding of the pathogenesis leading to keloid formation and suggest novel biomarkers and therapeutic strategies for keloids. Our findings may facilitate the development of novel targeted therapies for keloids.

### 2.3 Protein digestion and peptide purification

In‐solution digestion was performed before MS analysis. Twenty μg proteins were diluted to a final volume of 20 μL with digestion buffer (50 mM NH_4_HCO_3_, pH 8.0). The sample was treated with 1 μL of 1 M DTT, mixed well, and incubated at 50°C for 15 min, followed by the addition of 1 μL of 550 mM iodoacetamide for alkylation in darkness for 15 min at 25°C. Subsequently, 80 μL of digestion buffer and 1 μg of trypsin (Promega, Madison, USA) were added to the sample followed by incubation overnight at 37°C. The reaction was terminated by the addition of 33 μL of buffer (2% trifluoroacetic acid, 20% acetonitrile). Finally, the sample was purified by using C18 spin columns (Thermo Fisher Scientific, Rockford, USA) according to the manufacturer's instructions and dried with a vacuum centrifuge (Thermo Fisher Scientific, Asheville, USA).

### 2.4 Liquid chromatography (LC)‐mass spectrometry (MS)/MS analysis

The peptides were separately analyzed by using Q‐Exactive Orbitrap mass spectrometer (Thermo Fisher Scientific, Bremen, Germany) equipped with an EASY‐nLC 1200 (Thermo Fisher Scientific, Bremen, Germany). Data dependent acquisition (DDA) mode was applied. Precolumn (2 cm, 100 μm inner diameter, 5 μm C18 filler; Thermo Fisher Scientific, Bellefonte, USA) and analytical columns (10 cm, 75 μm inner diameter, 3 μm C18 filler; Thermo Fisher Scientific, Bellefonte, USA) were used for analysis. Peptides were eluted with a 90 min HPLC gradient from 0% to 100% in buffer (80% acetonitrile, 1% formic acid) at a flowrate of 250 nL/min. The scan range was set to 400–1700 *m*/*z*, and 70,000 resolution (at *m*/*z* 200) was used. The ten most‐abundant MS1 features were selected for high‐energy and MS/MS scans. Raw data were processed with Xcalibur software (Thermo Fisher Scientific, Bremen, Germany). The mass spectrometry proteomics data were deposited in the ProteomeXchange Consortium via the PRIDE partner repository with the dataset identifier PXD029631 [17].

### 2.5 Data analysis and quantification of proteomic raw files

MS raw files were analyzed by MaxQuant (version 1.6.12.0) with the UniProt reference protein database (Homo sapiens, November, 2019). The label‐free quantification (LFQ) algorithm was used for protein quantification. The option “*match between runs*” was applied to increase the number of peptide searches. Other options were set as default settings. Data arising from MaxQuant analysis was further processed with Microsoft excel.

### 2.6 RNA isolation and quantitative real‐time PCR (qRT‐PCR)

Total RNAs were extracted with the Trizol Reagent. Reverse transcription was performed with HiScript III 1st Strand cDNA Synthesis Kit (Vazyme, Nanjing, China). qRT‐PCR was carried out with the SYBR Green PCR Master Mix (Takara, Dalian, China). All processes were carried out in accordance with the manufacturer's protocol.

### 2.7 Western blot (WB)

Tissue sample was homogenized in RIPA lysis buffer with cocktail and centrifuged at 12,000 g for 10 min at 4°C. Supernatant was collected and the protein concentration was determined by Bradford assay. Twenty μg of proteins were used for WB analysis. Anti‐SPARC, anti‐RCN1, and anti‐CALU antibodies were obtained from Abcam. Anti‐PDGFRL and anti‐XBP1 antibodies were purchased from Cusabio and Sino Bioloqical separately.

### 2.8 PCA and heatmap analysis

Principal component analysis (PCA) and heatmap analysis were performed by the online tool ClustVis (http://biit.cs.ut.ee/clustvis/) [18]. Principal components were calculated by the default method in the R package using data that contained missing values. Heatmap analysis with a clustering tree was obtained via the heatmap tool in the R package.

### 2.9 Gene set enrichment analysis

The online Gene SeT AnaLysis Toolkit (WebGestalt) was used for gene set enrichment analysis by using the human genome reference gene set as a background which is freely available at http://www.webgestalt.org. Gene Ontology (GO), Kyoto Encyclopedia of Genes and Genomes (KEGG) analysis were performed by using the companion tool GOView. GO analysis was used to cluster the differently expressed proteins (*p *< 0.05) into three key biological aspects, including biological process, cellular component, and molecular function. The over‐representation of both GO and KEGG pathways was determined by the hypergeometric test. The *p*‐value was adjusted and calculated by Benjamini and Hochberg methods. Ingenuity pathway analysis (IPA) was performed to identify pathways involved in genes related to keloid formation.

### 2.10 Protein–protein interaction (PPI) network construction

The interaction between differently expressed proteins was illustrated by using the online tool for the Retrieval of Interacting Genes (STRING) online database (http://string‐db.org). The differential gene lists were submitted and searched by selecting “*Organism*” and “*Homo sapiens*” as key parameters. PPI networks were constructed based on the STRING database.

### 2.11 Human skin gene expression dataset

The gene expression data used in this study (GTEXv8 Human Skin‐Not Sun Exposed‐Suprapubic RNASEquation (Feb20) TPM log2) was accessed at the GeneNetwork (GN) website (http://genenetwork.org/). To investigate the genes that coexpressed with PDGFRL, we set the following parameters: “*Human (hg19)*” for Species, “*GTEx v8 All Tissues, RNA‐Seq without Genotypes*” for Group, “*Skin‐Not Sun Exposed (Suprapubic) mRNA*” for Type, “*GTEXv8 Human Skin‐Not Sun Exposed‐Suprapubic RNA‐SEquation (Feb20) TPM log2*” for Data Set, and entered “*PDGFRL*” for Get Any.

## 3 RESULTS

### 3.1 Differences in proteome profiling between keloid scar and normal skin tissues

**FIGURE 1:**
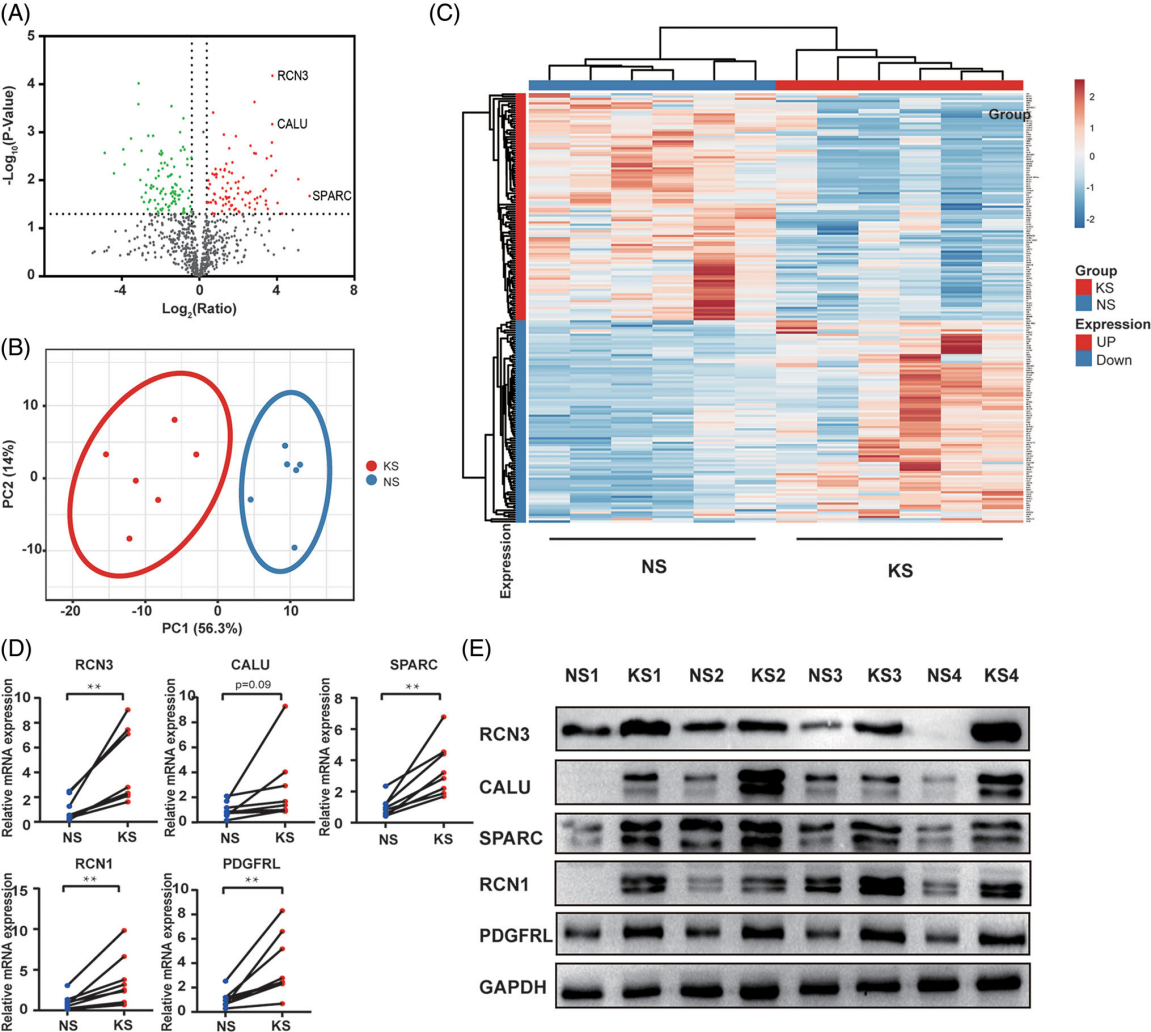
Differential proteomics analysis between keloid scar and normal skin tissues. (A) Volcano plot graph illustrating of differentially expressed proteins. The Y‑axis represents −log10 (*p* value) while the X‑axis represents log2 (ratio KS/NS). The up‐ and downregulated proteins in KS are indicated by red and green dots, respectively. (B) PCA plot of quantitative proteome profiles of KS and NS. Blue and red ellipses represent the clustering of samples. Clustering indicated the obvious differentiation of KS and NS groups. (C) Heatmap of quantitative proteome profiles of KS and NS samples. The up‐ and downregulated proteins are marked by red and blue, respectively. (D) The mRNA expression levels of SPARC, RCN3, RCN1, CALU, and PDGFRL were determined by qRT‐PCR. (E) The protein levels of SPARC, RCN3, RCN1, CALU, PDGFRL were determined by WB. Statistical analyses were performed by a two‐tailed paired Student's *t*‐test (**p < 0.01). KS, keloid scar; NS, normal skin; PCA, principal component analysis; qRT‐PCR, quantitative real‐time PCR

A total of 1359 proteins were identified and quantified from six pairs of keloid scars and adjacent normal skin tissue samples. Of these, 206 proteins exhibited a significant difference in expression between keloid scar and normal skin tissues. Ten proteins were uniquely identified in the keloid scar tissue samples and nine proteins in the normal skin samples (Supporting Information Table S2a and b). Eighty‐seven proteins were upregulated (*p *< 0.05, ratio > 1.3) and 100 proteins were downregulated (*p *< 0.05, ratio < 0.77) in keloid scar samples (Supporting Information Table S3a and b). A volcano plot was generated to highlight the distribution of these differentially proteins (Figure 1A). The PCA analysis separated the data into two subgroups: a keloid scar group and a normal skin group (Figure 1B). Cluster analysis also separated the proteins of all samples in each group (Figure 1C). The expression levels of potential biomarkers including SPARC, RCN1, RCN3, CALU, and PDGFRL were detected by qRT‐PCR and WB (Figure 1D, E). Of these, SPARC has been reported to play a role in scarring properties and ECM formation.

### 3.2 Biological, cellular, and molecular functional characteristics of the differentially expressed proteins

**FIGURE 2:**
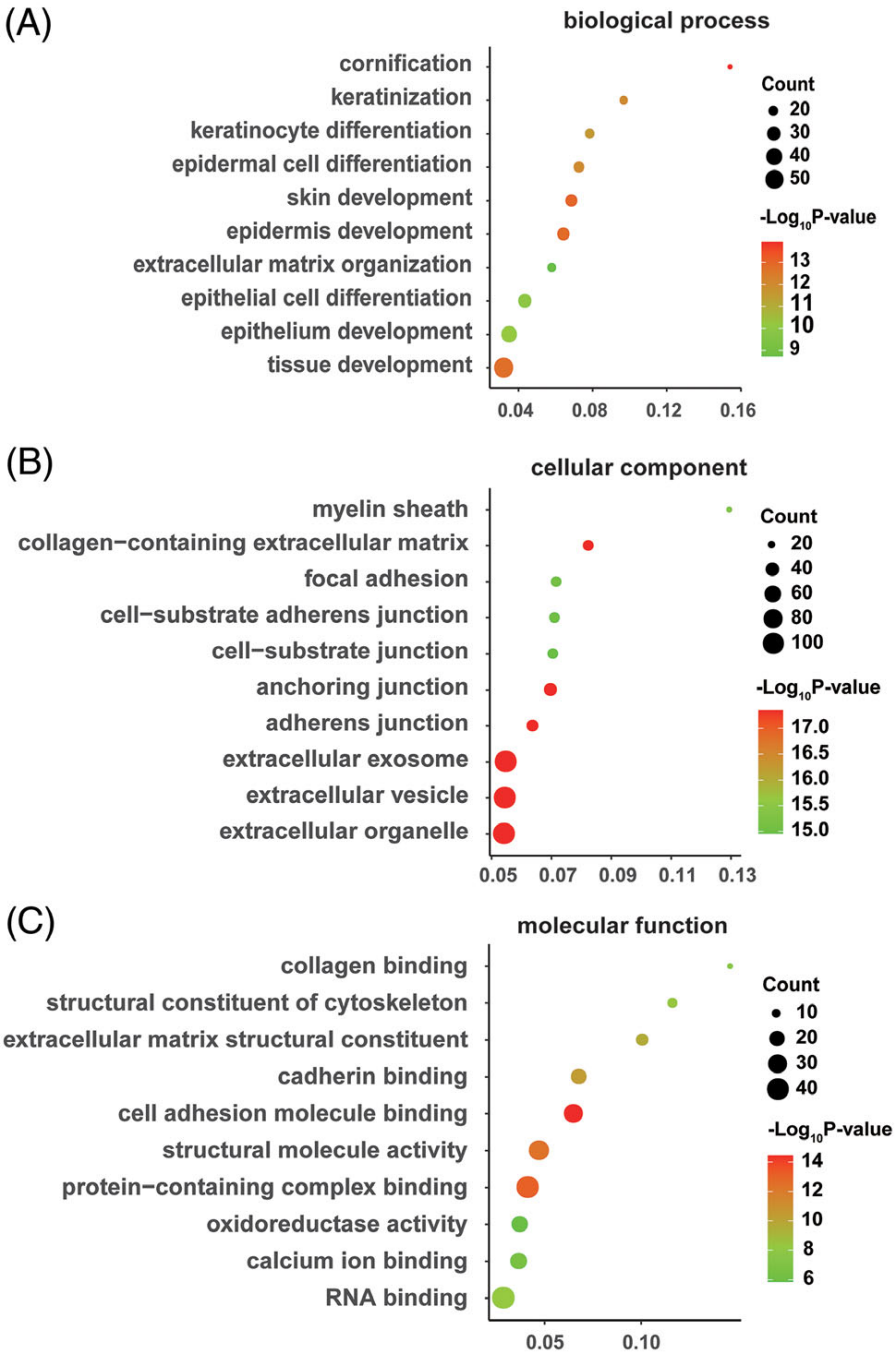
Enrichment analysis of differentially expressed proteins as determined by Gene Ontology (GO) analysis. (A) Biological process analysis. (B) Cellular component analysis. (C) Molecular function analysis. Color and size of the dots represent −log10 (*p* value) and the number of enriched genes, respectively

GO analyses relating to biological process, cellular component, and molecular function are shown in Figure 2. Variations in the differentially expressed proteins linked with biological processes were mainly enriched in cornification, keratinization, keratinocyte differentiation, epidermal cell differentiation, and skin development. With regards to cellular component, variations were significantly enriched in myelin sheath, collagen‐containing extracellular matrix, focal adhesion, cell‐substrate adherent junction, and cell‐substrate junction. Variations linked with molecular function were significantly enriched in collagen binding, structural constituent of cytoskeleton, extracellular matrix structural constituent, cadherin binding, and cell adhesion molecule binding.

### 3.3 PPI and module network construction

**FIGURE 3:**
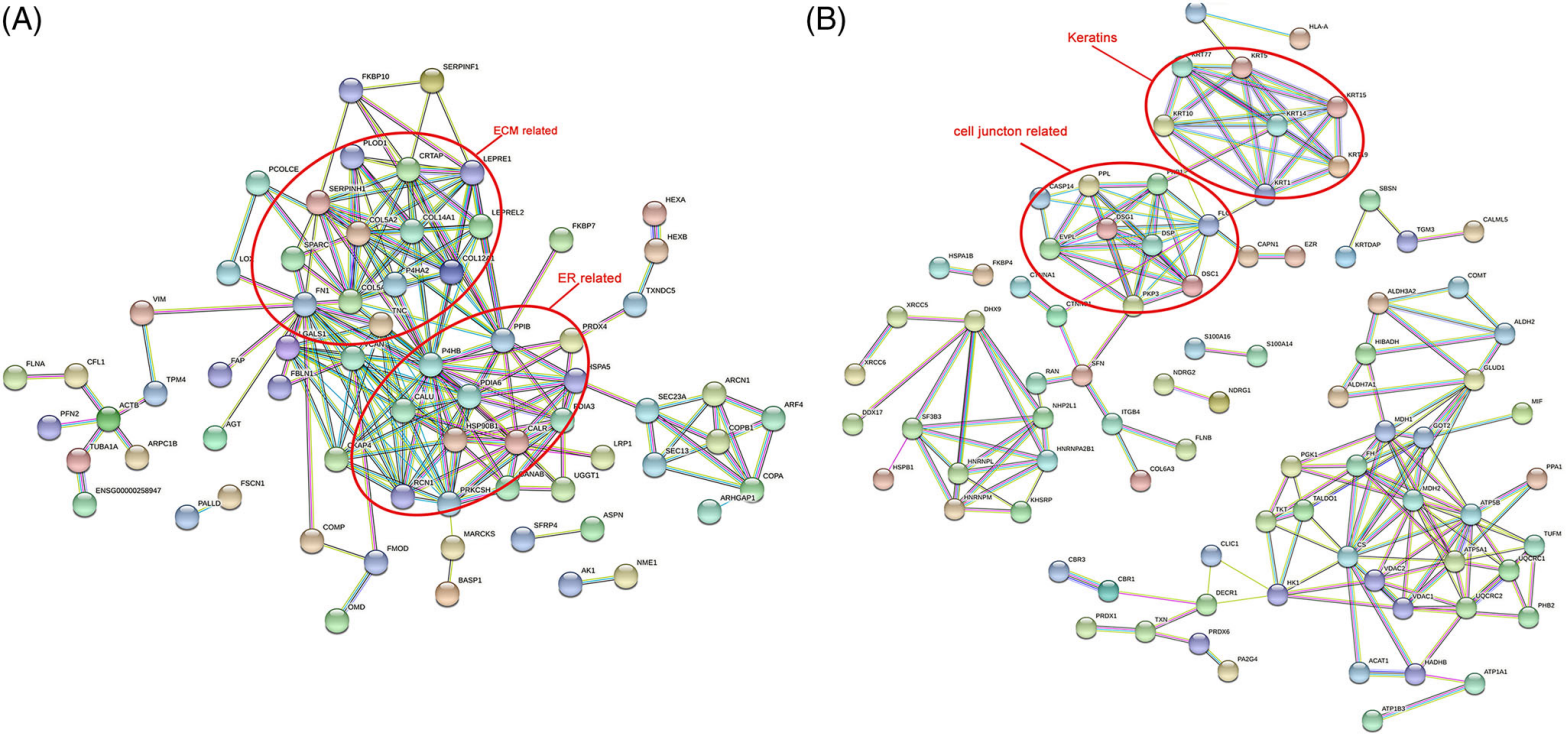
Protein–protein interaction (PPI) network of the differentially expressed proteins, as constructed by STRING. (A) PPI network of significantly upregulated proteins. (B) PPI network of significantly downregulated proteins

PPI network analysis was performed to illustrate the differentially expressed proteins (Figure 3). The PPI network showed that extracellular matrices such as COL5A1, COL5A2, COL12A1, COL14A1, COMP, TNC, FN1, LGALS1, and VCAN were upregulated in keloids. Some modifying enzymes, which play important roles in the formation of collagen, were also shown to have high expression levels in keloids, including P3H3, P3H4, PLOD1, and CRTAP. Most keratins were downregulated in keloids, including KRT10, KRT14, KRT15, KRT19, KRT1, KRT77, and KRT5. CASP14, a protein that is required for the cornification process, was also downregulated in keloids. Moreover, several proteins that play a role in cell junctions were also downregulated in keloids, including EVPL, PPL, DSP, PKP1, PKP3, DSC1, DSG1, and FLG.

### 3.4 Pathway analysis

**FIGURE 4:**
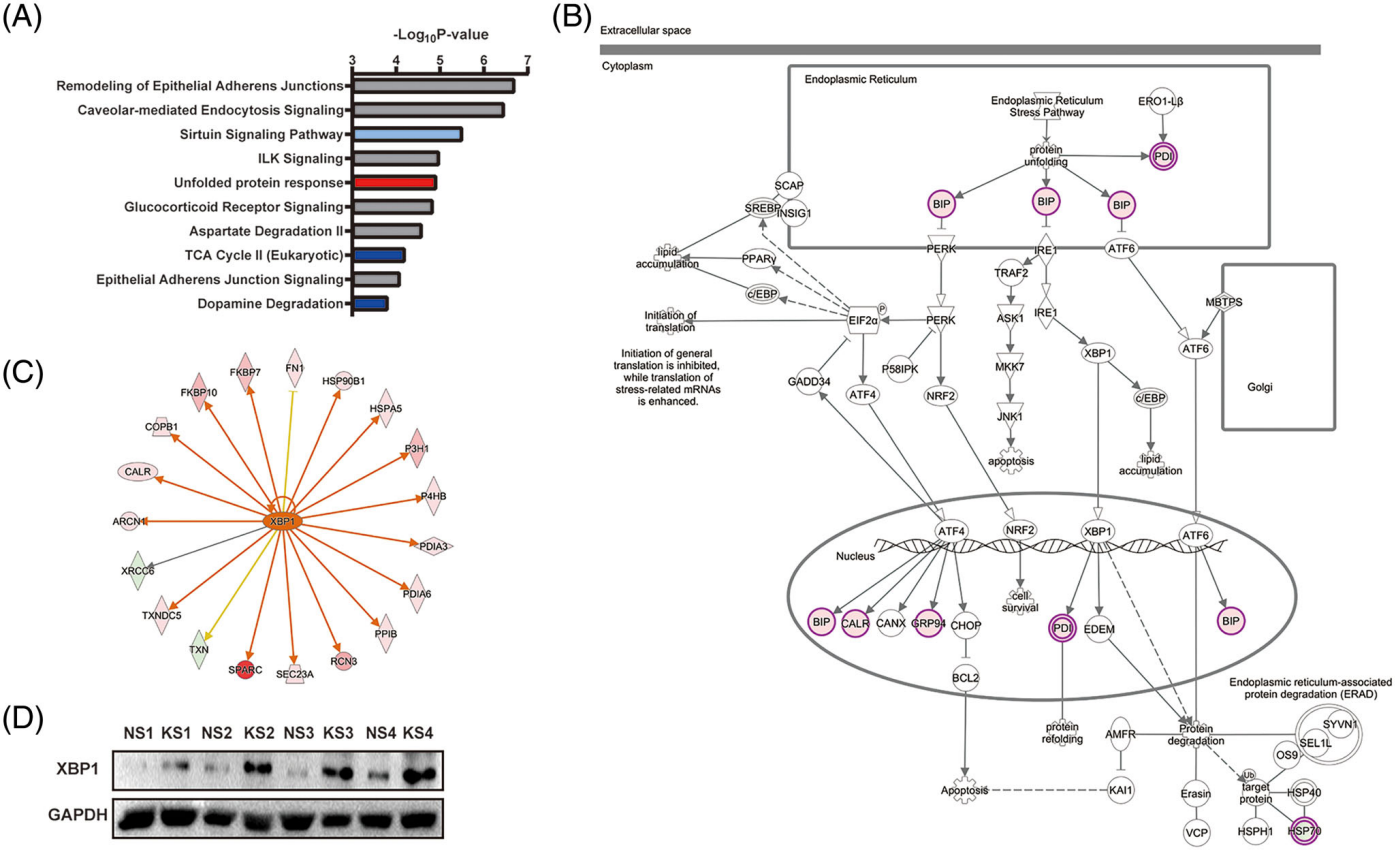
Pathway analysis of proteins showing significant alterations. (A) Pathway analysis of significantly differentially expressed proteins by IPA. The top 10 canonical pathways of the altered proteins were calculated by a right‐tailed Fisher's exact test. The red and blue colors represent significant activation and inhibition pathways, respectively. (B) The activated UPR pathway network in endoplasmic reticulum. This network showed that PDI, BIP, and CALR were upregulated and induced endoplasmic reticulum stress. (C) The XBP1 regulatory network was predicted by experimental evidence derived from the IPA database. (D) The protein level of XBP1 in keloid scars and normal skin tissues was determined by WB. IPA, ingenuity pathway analysis; KS, keloid scar; NS, normal skin; UPR, unfolded protein response; WB, western blot

To address the differentially expressed proteins enriched pathways, we performed IPA analysis. The top 10 related pathways are presented in Figure 4A. Pathway related to the unfolded protein response (UPR) function was significantly activated in keloids (Z score > 1.5, *p *= 1.20 × 10^−5^). A detailed gene list related to this pathway is given in Supporting Information Table S4. Further details of the UPR pathway are shown in Figure 4B, in which PDI, BIP, and CALR were upregulated and induced endoplasmic reticulum stress. Although the overexpression of XBP1, the regulator of the UPR pathway, was not detected in our proteomic analysis, our pathway analysis showed that XBP1 might be a potential therapeutic target for keloids because most proteins that were overexpressed in keloids can be regulated by XBP1 (Figure 4C). The upregulated expression level of XBP1 in keloids was further verified by WB (Figure 4D).

### 3.5 PDGFRL coexpressed gene analysis

**FIGURE 5:**
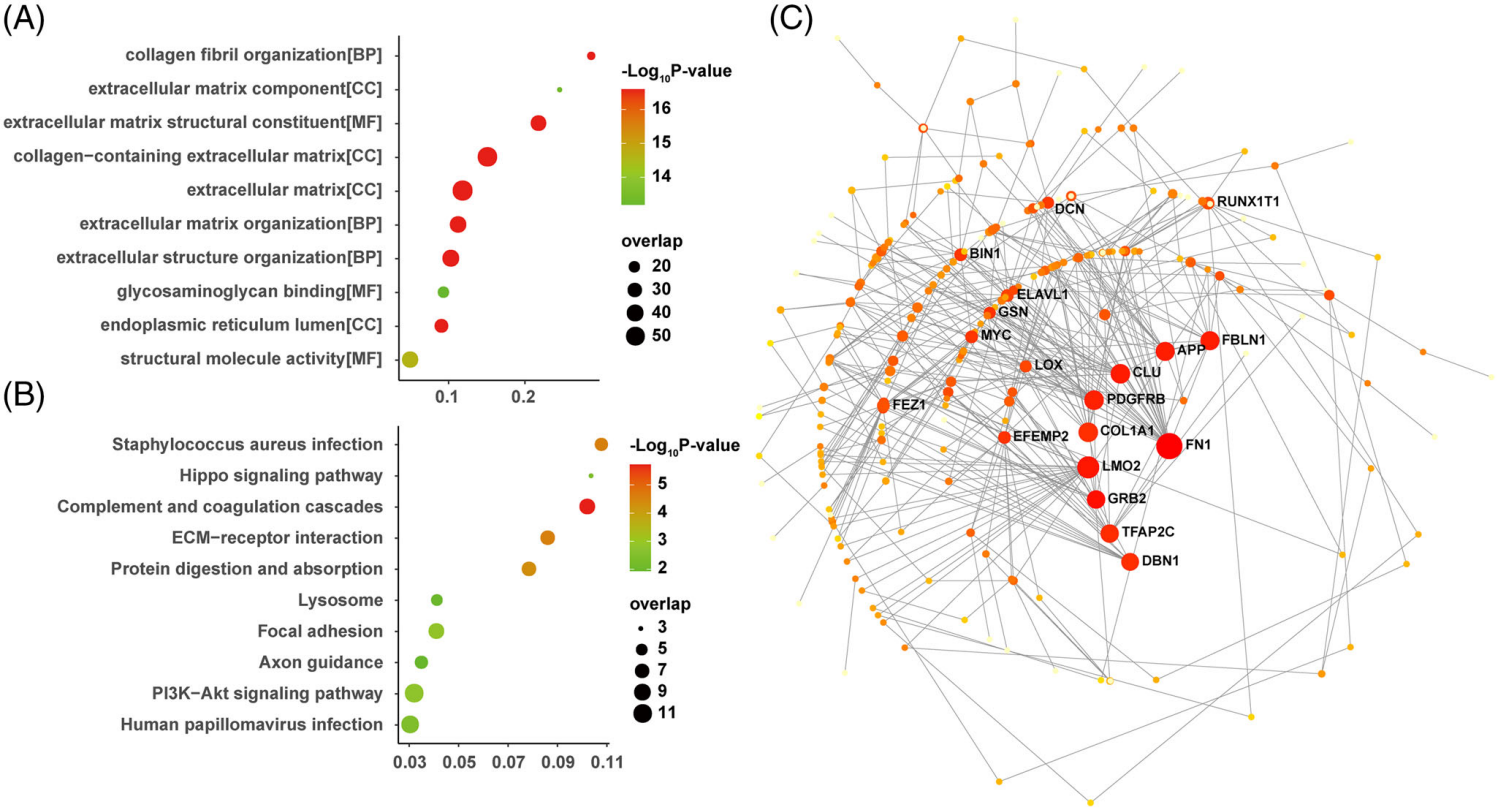
Genes that were coexpressed with PDGFRL, as determined by the GeneNetwork database. (A, B) The top 192 proteins that correlated with PDGFRL (*p* < 0.01, *r* > 0.7) were enriched by GO analysis and KEGG analysis. The X‐axis represents the enriched ratio while the Y‐axis represents the enriched terms or pathways. Color and size of the dots indicate −log10 (*p* value) and the number of enriched genes, respectively. (C) The construction of a PPI network predicted key regulators of PDGFRL. The nodes in the network represent the key genes. GO, Gene Ontology; KEGG, Kyoto Encyclopedia of Genes and Genomes; PPI, protein–protein interaction

PDGFRL was identified as one of the unique proteins expressed in keloid scar tissues. Considering that PDGFRL exhibits significant sequence similarity with the extracellular domain of PDGFR, which contributes to keloid formation, it follows that PDGFRL may also be involved in the keloid scarring. We performed genetic correlation analysis for *PDGFRL* against the skin transcriptome. The top 192 genes significantly correlated with the expression of *PDGFRL* (*p *< 0.01, *r* > 0.7) were further analyzed by GO function enrichment and KEGG pathway analysis (Figure 5A, B). The most related biological processes were collagen fibril organization and extracellular matrix organization. The most related cellular components were extracellular matrix component, collagen‐containing extracellular matrix, and extracellular matrix. The most related molecular function was extracellular matrix structural constituent. The top related pathways included *Staphylococcus aureus* infection, the Hippo signaling pathway, the complement and coagulation cascades, ECM–receptor interaction, protein digestion, and protein absorption. Next, the top 192 genes were also submitted to NetworkAnalyst software (www.networkanalyst.ca) so that we could analyze the PPI network with regards to curated and nonredundant sets of protein interactions in the IMEx consortium database [19]. As shown in Figure 5C, there were 10 proteins at the central node of the network which exhibited the highest degree of connections to other genes: *FN1*, *FBLN1*, *APP*, *CLU*, *DBN1*, *LMO2*, *GRB2*, *TFAP2C*, *PDGFRB*, and *COL1A1*.

## 4 DISCUSSION

In this study, we presented the most comprehensive proteomics study of keloids. Our results yielded a profile of 1359 proteins and identified 206 significantly differently expressed proteins between keloid scar and normal skin tissues. PPI analysis of the 206 proteins showed that most of the extracellular matrix proteins and ECM‐associated proteins were significantly upregulated in keloids including COL12A1, COL14A1, PLOD1, and SPARC. Most keratins and cell junction related proteins such as KRT10, EVPL, PPL, and DSP were downregulated in keloids. Our results were consistent with a previous study in which the matrisome (ECM components) of keloid and healthy skin tissue samples were investigated and characterized [20]. In both studies, the accumulation of collagens showed molecule‐specific (e.g., upregulated COL12A1 vs. downregulated COL6A3 in keloids). This means more special proteases for the collagen degradation are needed for the treatment of keloids. The fact that some proteoglycans related to skin mechanical forces (e.g., ASPN, VCAN, and OMD) were also overexpressed in keloids proved the interaction between skin mechanical disorder and keloid formation again.

Bioinformatics analysis indicated that pathways related to ER stress are enriched in the keloids. ER homeostasis is constantly challenged by a range of physiological activities, including Ca^2+^ reservoir regulation and the biosynthesis of lipids and sterols during physiological and pathophysiological perturbations. There are three major UPR response proteins: PERK, eIF2α, and IRE1α; these all bind to BiP, also known as HSPA5, in an inactive form under non‐ER stress conditions. In our research, we detected upregulated BiP and PDI, which help to correct the folding of proteins, including P4HB, PDIA3, and PDIA6 in keloids (Figure 4B). It seems that XBP1 plays an important role in keloid formation because most of the upregulated proteins were shown to be related with XBP1 (Figure 4C). For example, SPARC, a Ca^2+^‐binding glycoprotein, which exhibits increased level of expression in hypertrophic scars, has been reported to be upregulated by XBP‐1 in hepatocellular carcinoma cells [21, 22]. P3H1 and PPIB, which are essential for prolyl 3‐hydroxylation and folding of procollagens in the ER, have been shown to be upregulated by XBP1 in NIH‐3T3 fibroblasts [23]. PDI, chaperones, and COPI vesicles, which help protein folding and transport, have also been considered to be upregulated by XBP1, including P4HB, PDIA3, PDIA6, CALR, HSPA5, HSP90B1, FKBP7, FKBP10, COPB1, and ARCN1 [23]. Excisional wound healing is also related to increased level of the active form of XBP1 when compared with normal fibroblasts. Moreover, XBP1 along with the UPR signal pathway were activated in keloid fibroblasts when exposed to a hypoxic environment [24]. In another study, the inhibition of IRE1α also decreased scar formation and decreased XBP1 expression [25]. In a recent study, higher ER stress signaling was demonstrated in keloids than in normal tissues; this was consistent with our current results, and the inhibition of ER stress significantly decreased scar formation in a rabbit model [26]. These results suggested that XBP1, as a key regulator of UPR pathway, is closely related with keloid formation and may represent a potential therapeutic target for keloids.

Abnormal calcium homeostasis is associated with ER stress and has been reported to exist in keloid fibroblasts [27]. In our study, we found CALU, RCN3, and RCN1, the members of CREC protein family which carries out a number of functional activities, including calcium homeostasis and secretory cargo sorting, were significantly upregulated in keloids [28]. Of these, CALU and RCN3 showed abnormally high expression levels with lower *p* values in keloids (Figure 1A); while RCN1 was uniquely present in keloids (Supporting Information Table S2a). RCN1 is known to be able to suppress ER stress‐induced apoptosis and is related with tumorigenesis [29, 30, 31, 32]. CALU localizes to the entire secretory pathway, including the ER, Golgi apparatus, and the extracellular matrix. It appears that extracellular CALU inhibits cell migration, whereas nuclear isoform calumenin‐15 promotes filopodia formation and cell migration, which suggests that this protein exerts different functions when localized in different sites [33, 34]. RCN3 has been reported to be associated with the maturation of alveolar epithelial type II (AECII) cells during alveogenesis [35]. The expression of RCN3 in AECIIs appears to contribute to cell survival and wound healing [36]. The overexpression of RCN3 in keloids was also highlighted in a study of familial keloid [37]. These results indicated that CALU, RCN3, and RCN1 may be associated with keloid formation and could be novel potential biomarkers of keloids.

As one of the uniquely expressed proteins in keloid scars, PDGFRL could be considered as a potential candidate for the diagnosis and treatment of keloids. The precise biological function of PDGFRL is still under debate. Although it has been reported that PDGFRL inhibits the proliferation and invasion of colorectal cells; it has also been reported that PDGFRL contributes to the maintenance of the proliferating chondrocytes phenotype [38], thus indicating that PDGFRL plays diverse roles depending upon cell types. PPI network analysis identified 10 key regulators of the genes that were coexpressed with *PDGFRL*, including *FN1*, *FBLN1*, *APP*, *CLU*, *PDGFRB*, *COL1A1*, *LMO2*, *GRB2*, *TFAP2C*, and *DBN1*. In these proteins, TFAP2C is a transcription factor that is known to help organize ECM fibers [39]. LMO2 is known to regulate hematopoiesis and vascular development and has been reported to play an import role in tissue regeneration [40]. GBR2 is involved in the Ras signaling pathway and is linked to mitogenesis and cytoskeletal reorganization by EGF and PDGF [41]. DBN1, an actin‐binding protein, is involved in many cell–cell communication systems, including gap junctions and adherens junctions [42]. CLU, which performs a number of different functions and has also been associated with neurodegenerative diseases and cancer, also acts as a chaperone outside the cell and facilitates the extracellular clearance of misfolded proteins [43]. APP is mainly associated with Alzheimer's disease and participates in the control of epidermal wound repair [44]. As important components of the ECM, FBLN1, COL1A1, and FN1 are involved in ECM assembly, cell migration, and wound healing. They are also related to the proliferation and differentiation of osteoblasts [45, 46, 47, 48]. Considering that PDGFRL regulates the proliferation of chondrocytes, which share the same origin as osteoblasts, it follows that there may be potential interactions between FBLN1, COL1A1, FN1, and PDGFRL. The fact that we observed the upregulation of FBLN1, FN1, and PDGFRL in keloids in our proteomic profiles enhances the possibility of this hypothesis. We also identified PDGFRB in our network. However, the overexpression of PDGFRB was not detected in our study. PDGFRL may show a redundant function or a differential function with PDGFRB; thus, PDGFRL may also be a potential therapeutic target of keloids.

## 5 CONCLUDING REMARKS

In summary, we provided a comprehensive proteome profiling of keloid scars and normal skin tissues. We identified 206 proteins that showed significant differences in expression between keloid scars and normal skin tissues, including RCN3, RCN1, CALU, and PDGFRL which may play an import role in keloid formation. We proposed that the XBP1‐mediated UPR pathway plays a role in keloid formation. We also created a *PDGFRL* coexpression gene network to identify its potential functions. In summary, our study provides novel information relating to the pathological process of keloid formation and could contribute to the development of new therapeutic strategies for keloid scars.

## AUTHOR CONTRIBUTIONS

*Conceived the study*: Yibing Wang. *Performed the experiments*: Jian Liu, Chunhua Yang, Huayu Zhang, Wei Hu, and Xiaodong Chi. *Collected the samples*: Jian Liu and Huayu Zhang. *Performed data analysis*: Tingzhi Deng and Xiaodong Chi. *Wrote the manuscript, prepared the figures, and tables*: Jian Liu, Chunhua Yang, Huayu Zhang, Tingzhi Deng, Chao Zhang, and Xueling Yang. *Edited the manuscript*: Jonas Bergquist, Helen Wang, Jia Mi, Xiaodong Chi, and Yanping Zhu. All authors have read and approved the final manuscript.

## CONFLICT OF INTEREST

The authors have no conflict of interest to declare.

## Supporting information

Supporting Information

Supporting Information

Supporting Information

Supporting Information

## Data Availability

The mass spectrometry proteomics data have been deposited to the ProteomeXchange Consortium.

## References

[1] Ogawa, R. (2017). Keloid and hypertrophic scars are the result of chronic inflammation in the reticular dermis. International Journal of Molecular Sciences, 18(3), 606. 10.3390/ijms18030606 PMC5372622 28287424

[2] Berman, B. , Maderal, A. , & Raphael, B. (2017). Keloids and hypertrophic scars: Pathophysiology, classification, and treatment. Dermatologic Surgery, 43(1), S3–S18. 10.1097/DSS.0000000000000819 27347634

[3] Totan, S. , Echo, A. , & Yuksel, E. (2011). Heat shock proteins modulate keloid formation. Eplasty, 11, e21. 21559318 PMC3086522

[4] Tan, E. M. L. , Qin, H. , Kennedy, S. H. , Rouda, S. , Fox, J. W. , & Moore, J. H. Jr. (1995). Platelet‐derived growth factors‐AA and ‐BB regulate collagen and collagenase gene expression differentially in human fibroblasts. Biochemical Journal, 310(Pt 2), 585–588. 10.1042/bj3100585 7654198 PMC1135935

[5] Kischer, C. W. , & Pindur, J. (1990). Effects of platelet derived growth factor (PDGF) on fibronectin (FN) production by human skin and scar fibroblasts. Cytotechnology, 3(3), 231–238. 10.1007/BF00365486 22358773

[6] Thapa, R. K. , Margolis, D. J. , Kiick, K. L. , & Sullivan, M. O. (2020). Enhanced wound healing via collagen‐turnover‐driven transfer of PDGF‐BB gene in a murine wound model. ACS Applied Bio Materials, 3(6), 3500–3517. 10.1021/acsabm.9b01147 PMC7351314 32656505

[7] Mofikoya, B. O. , Adeyemo, W. L. , & Ugburo, A. O. (2012). An overview of biological basis of pathologic scarring. The Nigerian Postgraduate Medical Journal, 19(1), 40–45. 22430601

[8] Messadi, D. V. , Le, A. , Berg, S. , Huang, G. , Zhuang, W. , & Bertolami, C. N. (1998). Effect of TGF‐beta 1 on PDGF receptors expression in human scar fibroblasts. Frontiers in Bioscience, 3, a16–a22. 9450987 10.2741/A246

[9] Zhang, T. , Wang, X.‐F. , Wang, Z.‐C. , Lou, D. , Fang, Q.‐Q. , Hu, Y.‐Y. , Zhao, W.‐Y. , Zhang, L.‐Y. , Wu, L.‐H. , & Tan, W.‐Q. (2020). Current potential therapeutic strategies targeting the TGF‐beta/Smad signaling pathway to attenuate keloid and hypertrophic scar formation. Biomedicine & Pharmacotherapy, 129, 110287. 10.1016/j.biopha.2020.110287 32540643

[10] Ong, C. T. , Khoo, Y. T. , Mukhopadhyay, A. , Masilamani, J. , Do, D. V. , Lim, I. J. , & Phan, T. T. (2010). Comparative proteomic analysis between normal skin and keloid scar. British Journal of Dermatology, 162(6), 1302–1315. 10.1111/j.1365-2133.2010.09660.x 20128793

[11] Javad, F. , & Day, P. J. R. (2012). Protein profiling of keloidal scar tissue. Archives of Dermatological Research, 304(7), 533–540. 10.1007/s00403-012-1224-6 22407076

[12] Lee, J. H. , Shin, J. U. , Jung, I. , Lee, H. , Rah, D. K. , Jung, J. Y. , & Lee, W. J. (2013). Proteomic profiling reveals upregulated protein expression of hsp70 in keloids. BioMed Research International, 2013, 621538. 10.1155/2013/621538 24260741 PMC3821890

[13] Zhu, Y. , Zhang, C. , Xu, F. , Zhao, M. , Bergquist, J. , Yang, C. , Liu, X. , Tan, Y. , Wang, X. , Li, S. , Jiang, W. , Ong, Q. , Lu, L. , Mi, J. , & Tian, G. (2020). System biology analysis reveals the role of voltage‐dependent anion channel in mitochondrial dysfunction during non‐alcoholic fatty liver disease progression into hepatocellular carcinoma. Cancer Science, 111(11), 4288–4302. 10.1111/cas.14651 32945042 PMC7648023

[14] Zhang, Y. , Wang, D. , Li, M. , Wei, X. , Liu, S. , Zhao, M. , Liu, C. , Wang, X. , Jiang, X. , Li, X. , Zhang, S. , Bergquist, J. , Wang, B. , Yang, C. , Mi, J. , & Tian, G. (2018). Quantitative proteomics of TRAMP mice combined with bioinformatics analysis reveals that PDGF‐B regulatory network plays a key role in prostate cancer progression. Journal of Proteome Research, 17(7), 2401–2411. 10.1021/acs.jproteome.8b00158 29863873

[15] Liu, F. , Zhang, Y. , Men, T. , Jiang, X. , Yang, C. , Li, H. , Wei, X. , Yan, D. , Feng, G. , Yang, J. , Bergquist, J. , Wang, B. , Jiang, W. , Mi, J. , & Tian, G. (2017). Quantitative proteomic analysis of gastric cancer tissue reveals novel proteins in platelet‐derived growth factor b signaling pathway. Oncotarget, 8(13), 22059–22075. 10.18632/oncotarget.15908 28423550 PMC5400646

[16] Wei, X. , Zhang, Y. , Yu, S. , Li, S. , Jiang, W. , Zhu, Y. , Xu, Y. , Yang, C. , Tian, G. , Mi, J. , Bergquist, J. , Zhao, M. , & Song, F. (2018). PDLIM5 identified by label‐free quantitative proteomics as a potential novel biomarker of papillary thyroid carcinoma. Biochemical and Biophysical Research Communications, 499(2), 338–344. 10.1016/j.bbrc.2018.03.159 29574154

[17] Perez‐Riverol, Y. , Csordas, A. , Bai, J. , Bernal‐Llinares, M. , Hewapathirana, S. , Kundu, D. J. , Inuganti, A. , Griss, J. , Mayer, G. , Eisenacher, M. , Pérez, E. , Uszkoreit, J. , Pfeuffer, J. , Sachsenberg, T. , Yılmaz, Ş. , Tiwary, S. , Cox, J. , Audain, E. , Walzer, M. , & Vizcaíno, J. A. (2019). The PRIDE database and related tools and resources in 2019: Improving support for quantification data. Nucleic Acids Research, 47(D1), D442–D450. 10.1093/nar/gky1106 30395289 PMC6323896

[18] Metsalu, T. , & Vilo, J. (2015). ClustVis: A web tool for visualizing clustering of multivariate data using Principal Component Analysis and heatmap. Nucleic Acids Research, 43(W1), W566–W570. 10.1093/nar/gkv468 25969447 PMC4489295

[19] Xu, F. , Gao, J. , Munkhsaikhan, U. , Li, N. , Gu, Q. , Pierre, J. F. , Starlard‐Davenport, A. , Towbin, J. A. , Cui, Y. , Purevjav, E. , & Lu, L. (2020). The genetic dissection of Ace2 expression variation in the heart of murine genetic reference population. Frontiers in Cardiovascular Medicine, 7, 582949. 10.3389/fcvm.2020.582949 33330645 PMC7714829

[20] Zhang, S. , Liu, B. , Wang, W. , Lv, L. , Gao, D. , Chai, M. , Li, M. , Wu, Z. , Zhu, Y. , Ma, J. , & Leng, L. (2021). The “Matrisome” reveals the characterization of skin keloid microenvironment. FASEB Journal, 35(4), e21237. 10.1096/fj.202001660RR 33715180

[21] Arai, M. , Kondoh, N. , Imazeki, N. , Hada, A. , Hatsuse, K. , Kimura, F. , Matsubara, O. , Mori, K. , Wakatsuki, T. , & Yamamoto, M. (2006). Transformation‐associated gene regulation by ATF6alpha during hepatocarcinogenesis. FEBS Letters, 580(1), 184–190. 10.1016/j.febslet.2005.11.072 16364319

[22] Chavez‐Muñoz, C. , Hartwell, R. , Jalili, R. B. , Jafarnejad, S. M. , Lai, A. , Nabai, L. , Ghaffari, A. , Hojabrpour, P. , Kanaan, N. , Duronio, V. , Guns, E. , Cherkasov, A. , & Ghahary, A. (2012). SPARC/SFN interaction, suppresses type I collagen in dermal fibroblasts. Journal of Cellular Biochemistry, 113(8), 2622–2632. 10.1002/jcb.24137 22422640

[23] Wu, J. , Zhang, W. , Xia, L. , Feng, L. , Shu, Z. , Zhang, J. , Ye, W. , Zeng, N. , & Zhou, A. (2019). Characterization of PPIB interaction in the P3H1 ternary complex and implications for its pathological mutations. Cellular and Molecular Life Sciences, 76(19), 3899–3914. 30993352 10.1007/s00018-019-03102-8 PMC11105654

[24] Butler, P. D. , Wang, Z. , Ly, D. P. , Longaker, M. T. , Koong, A. C. , & Yang, G. P. (2011). Unfolded protein response regulation in keloid cells. Journal of Surgical Research, 167(1), 151–157. 10.1016/j.jss.2009.04.036 19631342 PMC2888625

[25] Boyko, T. V. , Bam, R. , Jiang, D. , Wang, Z. , Bhatia, N. , Tran, M. C. , Longaker, M. T. , Koong, A. C. , & Yang, G. P. (2017). Inhibition of IRE1 results in decreased scar formation. Wound Repair and Regeneration, 25(6), 964–971. 10.1111/wrr.12603 29316036 PMC5854534

[26] Kim, S. , Lee, S. E. , Yi, S. , Jun, S. , Yi, Y.‐S. , Nagar, H. , Kim, C.‐S. , Shin, C. , Yeo, M.‐K. , Kang, Y. E. , & Oh, S.‐H. (2021). Tauroursodeoxycholic acid decreases keloid formation by reducing endoplasmic reticulum stress as implicated in the pathogenesis of keloid. International Journal of Molecular Sciences, 19, 10765. 10.3390/ijms221910765 PMC8509846 34639105

[27] Yan, Z. , Zhang, W. , Xu, P. , Zheng, W. , Lin, X. , Zhou, J. , Chen, J. , He, Q.‐Y. , Zhong, J. , Guo, J. , Cheng, B. , & Wang, T. (2021). Phosphoproteome and biological evidence revealed abnormal calcium homeostasis in keloid fibroblasts and induction of aberrant platelet aggregation. Journal of Proteome Research, 20(5), 2521–2532. 10.1021/acs.jproteome.0c00984 33710899

[28] Honoré, B. (2009). The rapidly expanding CREC protein family: Members, localization, function, and role in disease. Bioessays, 31(3), 262–277. 19260022 10.1002/bies.200800186

[29] Short, S. P. , Barrett, C. W. , Stengel, K. R. , Revetta, F. L. , Choksi, Y. A. , Coburn, L. A. , Lintel, M. K. , Mcdonough, E. M. , Washington, M. K. , Wilson, K. T. , Prokhortchouk, E. , Chen, X. , Hiebert, S. W. , Reynolds, A. B. , & Williams, C. S. (2019). Kaiso is required for MTG16‐dependent effects on colitis‐associated carcinoma. Oncogene, 38(25), 5091–5106. 10.1038/s41388-019-0777-7 30858547 PMC6586520

[30] Giribaldi, G. , Barbero, G. , Mandili, G. , Daniele, L. , Khadjavi, A. , Notarpietro, A. , Ulliers, D. , Prato, M. , Minero, V. G. , Battaglia, A. , Allasia, M. , Bosio, A. , Sapino, A. , Gontero, P. , Frea, B. , Fontana, D. , & Destefanis, P. (2013). Proteomic identification of Reticulocalbin 1 as potential tumor marker in renal cell carcinoma. Journal of Proteomics, 91, 385–392. 10.1016/j.jprot.2013.07.018 23916412

[31] Uzozie, A. C. , Selevsek, N. , Wahlander, A. , Nanni, P. , Grossmann, J. , Weber, A. , Buffoli, F. , & Marra, G. (2017). Targeted proteomics for multiplexed verification of markers of colorectal tumorigenesis. Molecular & Cellular Proteomics, 16(3), 407–427. 10.1074/mcp.M116.062273 28062797 PMC5341002

[32] Xu, S. , Xu, Y. , Chen, L. , Fang, Q. , Song, S. , Chen, J. , & Teng, J. (2017). RCN1 suppresses ER stress‐induced apoptosis via calcium homeostasis and PERK‐CHOP signaling. Oncogenesis, 6(3), e304. 10.1038/oncsis.2017.6 28319095 PMC5533947

[33] Feng, H. , Chen, L. , Wang, Q. , Shen, B. , Liu, L. , Zheng, P. , Xu, S. , Liu, X. , Chen, J. , & Teng, J. (2013). Calumenin‐15 facilitates filopodia formation by promoting TGF‐beta superfamily cytokine GDF‐15 transcription. Cell Death & Disease, 4, e870–e870. 10.1038/cddis.2013.403 24136234 PMC3920949

[34] Zheng, P. , Wang, Q. , Teng, J. , & Chen, J. (2015). Calumenin and fibulin‐1 on tumor metastasis: Implications for pharmacology. Pharmacological Research, 99, 11–15. 10.1016/j.phrs.2015.05.001 25976680

[35] Jin, J. , Li, Y. , Ren, J. , Man Lam, S. , Zhang, Y. , Hou, Y. , Zhang, X. , Xu, R. , Shui, G. , & Ma, R. Z. (2016). Neonatal respiratory failure with retarded perinatal lung maturation in mice caused by reticulocalbin 3 disruption. American Journal of Respiratory Cell and Molecular Biology, 54(3), 410–423. 10.1165/rcmb.2015-0036OC 26252542

[36] Jin, J. , Shi, X. , Li, Y. , Zhang, Q. , Guo, Y. , Li, C. , Tan, P. , Fang, Q. , Ma, Y. , & Ma, R. Z. (2018). Reticulocalbin 3 deficiency in alveolar epithelium exacerbated bleomycin‐induced pulmonary fibrosis. American Journal of Respiratory Cell and Molecular Biology, 59(3), 320–333. 10.1165/rcmb.2017-0347OC 29676583

[37] (2017). The application of iTRAQ quantitative proteomics in familial keloid. Zhonghua Zheng Xing Wai Ke Za Zhi = Zhonghua Zhengxing Waike Zazhi = Chinese Journal of Plastic Surgery, 33(2), 122–128. 30070812

[38] Kawata, K. , Kubota, S. , Eguchi, T. , Aoyama, E. , Moritani, N. H. , Oka, M. , Kawaki, H. , & Takigawa, M. (2017). A tumor suppressor gene product, platelet‐derived growth factor receptor‐like protein controls chondrocyte proliferation and differentiation. Journal of Cellular Biochemistry, 118(11), 4033–4044. 10.1002/jcb.26059 28407304

[39] Park, D. , Wershof, E. , Boeing, S. , Labernadie, A. , Jenkins, R. P. , George, S. , Trepat, X. , Bates, P. A. , & Sahai, E. (2020). Extracellular matrix anisotropy is determined by TFAP2C‐dependent regulation of cell collisions. Nature Materials, 19(2), 227–238. 10.1038/s41563-019-0504-3 31659294 PMC6989216

[40] Meng, S. , Matrone, G. , Lv, J. , Chen, K. , Wong, W. T. , & Cooke, J. P. (2016). LIM domain only 2 regulates endothelial proliferation, angiogenesis, and tissue regeneration. Journal of the American Heart Association, 5(10), e004117. 10.1161/JAHA.116.004117 27792641 PMC5121509

[41] Matuoka, K. , Shibasaki, F. , Shibata, M. , & Takenawa, T. (1993). Ash/Grb‐2, a SH2/SH3‐containing protein, couples to signaling for mitogenesis and cytoskeletal reorganization by EGF and PDGF. EMBO Journal, 12(9), 3467–3473. 10.1002/j.1460-2075.1993.tb06021.x 8253073 PMC413623

[42] Shirao, T. , & Sekino, Y. (2017). General introduction to drebrin. Advances in Experimental Medicine and Biology, 1006, 3–22. 10.1007/978-4-431-56550-5_1 28865011

[43] Rodríguez‐Rivera, C. , Garcia, M. M. , Molina‐Álvarez, M. , González‐Martín, C. , & Goicoechea, C. (2021). Clusterin: Always protecting. Synthesis, function and potential issues. Biomedicine & Pharmacotherapy, 134, 111174. 10.1016/j.biopha.2020.111174 33360046

[44] Kummer, C. , Wehner, S. , Quast, T. , Werner, S. , & Herzog, V. (2002). Expression and potential function of beta‐amyloid precursor proteins during cutaneous wound repair. Experimental Cell Research, 280(2), 222–232. 10.1006/excr.2002.5631 12413888

[45] Yang, C. , Wang, C. , Zhou, J. , Liang, Q. , He, F. , Li, F. , Li, Y. , Chen, J. , Zhang, F. , Han, C. , Liu, J. , Li, K. , & Tang, Y. (2020). Fibronectin 1 activates WNT/beta‐catenin signaling to induce osteogenic differentiation via integrin beta1 interaction. Laboratory Investigation, 100(12), 1494–1502. 10.1038/s41374-020-0451-2 32561820

[46] Cooley, M. A. , Harikrishnan, K. , Oppel, J. A. , Miler, S. F. , Barth, J. L. , Haycraft, C. J. , Reddy, S. V. , & Scott Argraves, W. (2014). Fibulin‐1 is required for bone formation and Bmp‐2‐mediated induction of Osterix. Bone, 69, 30–38. 10.1016/j.bone.2014.07.038 25201465 PMC4385289

[47] Hang Pham, L. B. , Yoo, Y.‐R. , Park, S. H. , Back, S. A. , Kim, S. W. , Bjørge, I. , Mano, J. , & Jang, J.‐H. (2017). Investigating the effect of fibulin‐1 on the differentiation of human nasal inferior turbinate‐derived mesenchymal stem cells into osteoblasts. Journal of Biomedical Materials Research Part A, 105(8), 2291–2298. 10.1002/jbm.a.36095 28445604

[48] Zhytnik, L. , Maasalu, K. , Pashenko, A. , Khmyzov, S. , Reimann, E. , Prans, E. , Kõks, S. , & Märtson, A. (2019). COL1A1/2 pathogenic variants and phenotype characteristics in Ukrainian osteogenesis imperfecta patients. Frontiers in Genetics, 10, 722. 10.3389/fgene.2019.00722 31447884 PMC6696896

